# Temporal–Spatial Surface Seasonal Mass Changes and Vertical Crustal Deformation in South China Block from GPS and GRACE Measurements

**DOI:** 10.3390/s18010099

**Published:** 2017-12-31

**Authors:** Meilin He, Wenbin Shen, Yuanjin Pan, Ruizhi Chen, Hao Ding, Guangyi Guo

**Affiliations:** 1 School of Geodesy and Geomatics, Wuhan University, Wuhan 430079, China; hemeilin269@163.com (M.H.); dhaosgg@sgg.whu.edu.cn (H.D.); 2 State Key Laboratory of Information Engineering in Surveying, Mapping and Remote Sensing, Wuhan University, Wuhan 430079, China; pan_yuanjin@163.com (Y.P.); ruizhi.chen@whu.edu.cn (R.C.); guangyi.guo@whu.edu.cn (G.G.)

**Keywords:** CGPS time series, GRACE observations and surface loads, empirical orthogonal function, vertical crustal deformation

## Abstract

The solid Earth deforms elastically in response to variations of surface atmosphere, hydrology, and ice/glacier mass loads. Continuous geodetic observations by Global Positioning System (CGPS) stations and Gravity Recovery and Climate Experiment (GRACE) record such deformations to estimate seasonal and secular mass changes. In this paper, we present the seasonal variation of the surface mass changes and the crustal vertical deformation in the South China Block (SCB) identified by GPS and GRACE observations with records spanning from 1999 to 2016. We used 33 CGPS stations to construct a time series of coordinate changes, which are decomposed by empirical orthogonal functions (EOFs) in SCB. The average weighted root-mean-square (WRMS) reduction is 38% when we subtract GRACE-modeled vertical displacements from GPS time series. The first common mode shows clear seasonal changes, indicating seasonal surface mass re-distribution in and around the South China Block. The correlation between GRACE and GPS time series is analyzed which provides a reference for further improvement of the seasonal variation of CGPS time series. The results of the GRACE observations inversion are the surface deformations caused by the surface mass change load at a rate of about −0.4 to −0.8 mm/year, which is used to improve the long-term trend of non-tectonic loads of the GPS vertical velocity field to further explain the crustal tectonic movement in the SCB and surroundings.

## 1. Introduction

The mass surface of the Earth is an elastomer, susceptible to some physical phenomena on the surface of the Earth. For example, the atmosphere, hydrology and non-tidal ocean mass redistribution will contribute to a flexible deformation of regional crust [1]. Changes in the mass of water, atmosphere and non-tidal ocean during climate cycles perturb the Earth’s gravity field following Newton’s law of gravitation, and the accompanying loading effects on the Earth surface deform the lithosphere [2]. With the development of space satellite technology, we have some in-depth understanding of Earth’s physical phenomena from surface water resources to deep crust dynamics. As the current field of Earth science observations, Global Positioning System (GPS) and Gravity Recovery and Climate Experiment (GRACE) technology provide us with a variety of trusted data sources. GPS observations present the displacement of the Earth’s crustal deformation, including seasonal changes and long-term trend tectonic motion in the continuous GPS observation stations [3,4]. The launched GRACE gravity satellites, as a means of changing gravity, provide us with an inversion of surface mass changes. Many studies have demonstrated that it is feasible to analyze regional seasonal changes by using GPS and GRACE observations [5,6]. Furthermore, regional deep crustal deformation and tectonic dynamic processes have been constrained by GPS and GRACE data [7,8,9].

The Southern China block (SCB) is located in the southeastern margin of Sichuan and Yunnan block, where the crustal movement and tectonic fracture play a great role in the development of crustal uplift. The SCB is one of the most diversified continental pieces that constitute the Eurasian continent. In addition, the SCB is one of the main continental pieces derived from Gondwana that assembled together with Siberia to form the present Eurasia [10]. The marine deposition in history was concluded in the South China continent and turned into the intra-continental tectonic development at the end of Middle Triassic Indosinian movement. Although not covering all the tectonic and geodynamic aspects experienced by this block, the contributions gathered in this region will shed light on the evolution of this complex continent. Many studies have focused on the Phanerozoic evolution of the SCB [11,12,13]. However, there are few research achievements about the present-day vertical crustal dynamic processing in and around the SCB.

In this study, we present a new vertical crustal deformation in South China and surroundings derived by regional continuous GPS and GRACE observations. The GPS time series for all sites were examined and preprocessed with the common mode errors (CME) to improve the signal-to-noise ratio by using the empirical orthogonal decomposition (EOF) method. The spatial seasonal vibrations and vertical tectonic movement of the SCB were constrained by improved CGPS and GRACE-RL05 spherical harmonic coefficients (the GRACE products). The differences between the GRACE inversion of the surface mass change and the spatial scale of the GPS observations are discussed. The vertical deformation of the SCB crust is calculated by deducting the long-term contributions of the GRACE-modeled load deformation from the GPS vertical field.

## 2. Data and Methods

### 2.1. GPS Dataset and Data Processing

**Figure 1:**
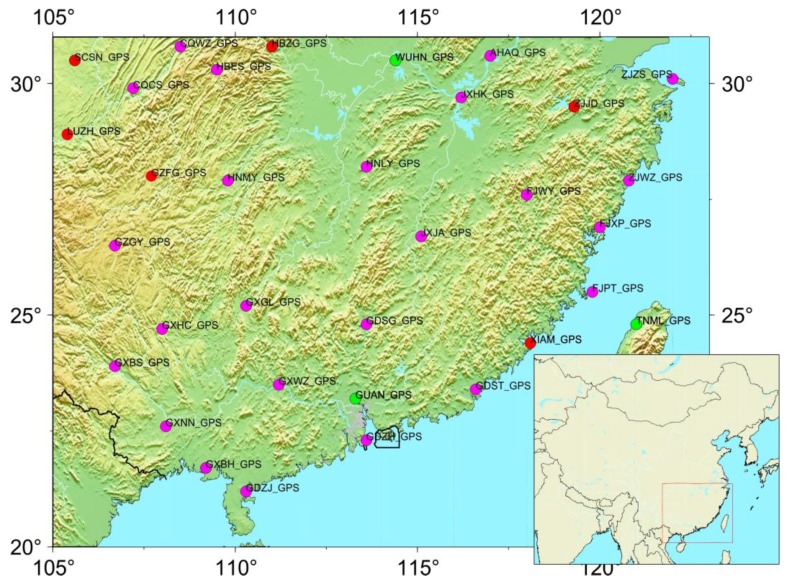
The distribution of GPS stations in and around the South China block (SCB). The magenta circles are the CGPS stations with records spanning from March 2010 to July 2016, and the green dots are the CGPS stations with records spanning from January 1999 to July 2016. The red dots are selected for comparison with GRACE-inferred loading displacement. The map in the inset shows the location of the SCB.

In this study, the used continuous and campaign-mode GPS data are primarily from the Crustal Movement Observation Network of China (CMONOC I and II). Most new CGPS stations from CMONOC II have started observation in 2010, and long-term observation stations from CMONOC I with recorded data from more than 16 years, the span from 1999 to 2016. We selected continuous GPS stations from China’s continental environmental monitoring network in the SCB, of which 5 GPS sites (GUAN, LUZH, TNML, WUHN, XIAM) during the span from 1999 to 2016, and other 28 GPS sites during the span from 2010 to 2016. The data recordings of each GPS stations time-span are more than 3 years. The distribution of GPS sites is shown in Figure 1, and details are given in Table 1.

The specific processing strategies setting for GPS data from (1) to (3) are as follows:

(1) GAMIT/GLOBK software for baseline calculation was used, combined with BJFS, LHAZ, WUHN, SHAO, KUNM, TNML, URUM, TASH, XIAA, from IGS stations in the Asian region, and was solved by the single day relaxation method [14]. The correction models used mainly include troposphere (Graphical Modeling Framework, GMF), ionosphere (Global Pressure and Temperature, GPT) [15,16], the ocean tide model (FES2004) and the IERS2003 Earth tide model [17]. We applied International Earth Rotation and Reference Systems (IERS) 2010 conventions to correct the tidal solid Earth and pole tides [17].
(2) GLOBK software was used to adjust the baseline to obtain GPS time series. The H-file of single day solution was jointed global subnet IGS1/IGS2/IGS3, as a benchmark, we selected core stations from International GNSS Service (IGS), such as VILL, KIT3, FORT, BRMU, GRAZ, PERT, YELL, LHAZ, SHAO, METS, TROM, CAS1, MATE, KOSG [18,19]. The IGS service website, supplied by the Scripps Orbital and Position Analysis Center (SOPAC, http://sopac.ucsd.edu/). The loosely constrained solution of the complete network was then aligned by a weighted six-parameter transformation (three translation and three rotation parameters) into the 2008 International Terrestrial Reference System (ITRF2008) reference frame [20,21].
(3) There are gaps and outliers (data with unsatisfactory results) in the CGPS time series, while with some noises, such as common mode errors in the regional GPS network, special data preprocessing for initial time series is needed. Here, we linearly interpolated the gaps using the averaging of neighbor values, and removed outliers by using an average smooth filter with a bandwidth of 10. Finally, we used the Quali-Observation Combination Analysis (QOCA) and the principal component analysis (PCA) program to preprocess the CGPS time series [22].

**Figure 2:**
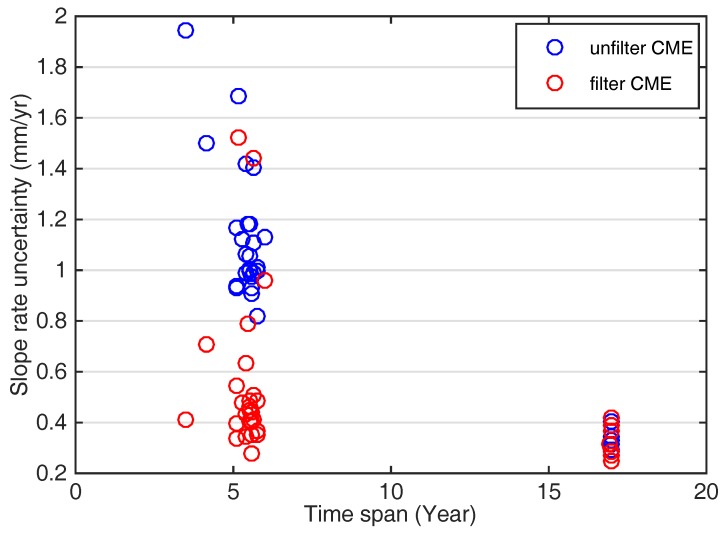
The slope rate uncertainty of common mode errors (CME) of GPS vertical velocity, blue dots are uncertainty with CME retained; red dots are with CME filtered.

The common mode errors (CME) have a certain influence on the long-term trend of the GPS-derived velocity due to the nonlinear signals in GPS time series. We analyzed their influence here and compared the uncertainty of the precision before and after the error elimination. Then, a white noise plus flicker noise model by a maximum likelihood estimation (MLE) using CATS software (version 3.1.2, Manufacturer by Simon Williams, Liverpool, UK) was applied to estimate the velocities and the associated realistic uncertainties for all continuous GPS stations [23,24]. The final slope rates were estimated with the annual and semi-annual signals added. The uncertainties of all GPS sites with common mode errors (CME) were retained and filtered respectively, as shown in Figure 2.

The observation time for GPS sites should be more than three years in order to obtain more reliable results of the crustal deformation with higher precision. It can be seen clearly that GPS time series with common mode errors filtered may significantly improve the accuracy of the slope rate uncertainty, as shown in Figure 2. In addition, the uncertainty of GPS time series will decrease with the increase of the integrated observing time, implying that the common mode errors may hardly have influence on the GPS velocity field with an observation time span of more than 16 years.

### 2.2. GRACE Model Data and Load Deformation Calculation

GRACE utilizes a state-of-the-art technique to observe variations of Earth’s gravity by tracking the inter-satellite range and range-rate between two coplanar, low altitude satellites via a K-band ranging (KBR) system [25]. Large-scale mass redistribution in and around the China continent has been monitored at a spatial resolution of approximately 100 km constrained from monthly GRACE gravity changes since April 2002, especially the inversion and application of surface water resources [26,27,28]. At such a spatial scale, monthly mass variations are determined with an accuracy of 1–2 cm in equivalent water height (EWH) [29]. In this study, we used the latest GRACE-RL05 products released by the Center for Space Research (CSR), University of Texas. Spherical harmonic coefficients up to a degree and order of 60 for the gravity field are provided monthly from April 2002 to January 2016. Among them, due to the GRACE spherical harmonic coefficients, especially low order terms of C20 cannot be accurately obtained. The Satellite Laser Ranging (SLR) estimation results were adopted for replacement [30] and the degree-1 coefficients given by Swenson et al. [31] were also used.

**Figure 3:**
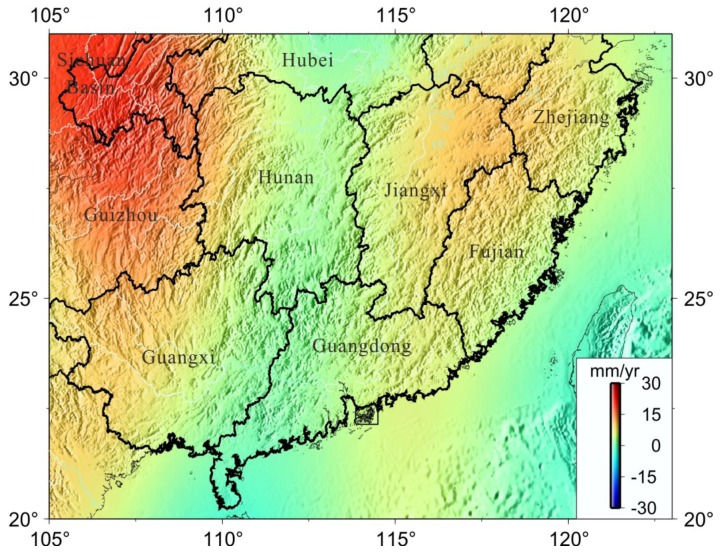
The GRACE-derived mass change (equivalent water thickness) in South China, time spanning from April 2002 to January 2016. The mass change in unit square volume is expressed as equivalent water thickness (mm/year).

Level-2 data products include various spherical harmonic coefficients such as geoid model (GSM), atmospheric model (GAA), ocean model (GAB), global atmospheric and oceanic model (GAC), and atmospheric ocean model, only in oceans (GAD) models. The solution of GSM products has been deducted from the non-tidal ocean atmosphere, the high frequency signal, tide and pole tide signal, so it mainly reflects the change of the gravity signal caused by the redistribution of surface water resources. Due to the fact that the GPS data contain the non-tidal and atmospheric loading effects, we used the CSR-provided De-aliasing Level-1B (AOD1B) solution (GAC model) and the monthly Stokes coefficient GSM (GRACE Satellite observed data) to compute the whole surface vertical mass loads, including the hydrological, atmospheric and non-tidal ocean loading effects. Therefore, the presented GPS-derived and GRACE-inferred time series are mainly consistent in surface mass loading signals. According to previous studies, the surface equivalent water height (EWH) can be expressed in terms of the Stokes coefficients as [32,33].

(1) $$\Delta \sigma (\phi ,\theta )=\frac{a\rho_{e}}{3\rho_{w}}\sum_{n=0}^{\infty }\left(\frac{2n+1}{1+k_{n}}\right)\sum_{m=0}^{n}\left\{\left[\Delta C_{n}^{m}\cos(m\phi )+\Delta S_{n}^{m}\sin(m\phi )\right]{\bar{P}}_{n}^{m}(\cos\theta )\right\}$$

where $\rho_{e}$ and $\rho_{w}$ are the average density of the whole Earth and the density of water (1 g/cm^3^), respectively; parameter a is the equatorial radius; $k_{n}$ is the load love number at degree n (n ≤ 200); ϕ is the latitude; θ is the colatitude; and $\Delta C_{n}^{m}$ and $\Delta S_{n}^{m}$ are monthly Stokes coefficients anomaly. ${\bar{P}}_{n}^{m}(\cos\theta )$ is the fully normalized Legendre function of degree n and order m. We employed global forward modeling to remove leakage biases in the GRACE-estimated mass changes due to truncation and spatial filtering and recover the true magnitudes of the signals, at least on a regional average basis. The EWH secular variations in and around Southern China are as shown in Figure 3. Note that GRACE-inferred surface e mass changes, including soil moisture (SM), canopy (C), river run-off (RR) and snow fall (SF) [34] will be further used to derive surface loading displacements corresponding to GPS sites location.

Figure 3 presents the spatial pattern of total secular water storage variations throughout South China. Most humid and rainy regions including Sichuan Basin, Guizhou and Jiangxi showed a clear positive signal reveal that the water increases at rates from 10 mm/year to 30 mm/year from 2002 to 2016. The increase of surface water resources is significantly correlated with the convergence of the water vapor transport and deep convections in South China [35,36]. The rains were abnormally heavy in the Yangtze basin, with 15 percent more rainfall in 2010 than in an average year [37]. Furthermore, inter-decadal variations in the early (May–June) summer monsoon rainfall over South China (SCMR) are related to the ENSO (El Nino/Southern Oscillation) events [38]. Therefore, the GRACE-derived total water storage is mainly caused by surface monsoon rainfall, which is seen as the driving force for regional soil moisture.

Mass variations and their balances will deform the Earth’s surface because the Earth is an elastic body [3]. Surface water resources, atmosphere and non-tidal ocean loads contribute to this deformation, especially to the vertical crustal non-tectonic movement. The global monthly spherical harmonic coefficients can be used to constrain the gravity change caused by water variations which can be identified by the vertical surface loading deformation. In data processing, due to the quality of spherical harmonic coefficients, GRACE-RL05 has been improved. Meanwhile, we used GRACE data only by 300 km Gaussian smoothing to reduce the impact of spherical harmonic coefficients of higher order noise. Finally, we obtained the time sequence corresponding to the position of loading displacement through the global grid interpolation. The vertical deformation caused by mass load changes can be expressed by the spherical harmonic function of the Earth gravity field and load love numbers:

(2) $$\Delta h(\theta ,\phi )=R\sum_{l=1}^{\infty }\sum_{m=0}^{l}{\bar{P}}_{lm}(\cos\theta )\cdot [C_{lm}\cos(m\phi )+S_{lm}\sin(m\phi )]\cdot \frac{h_{l}^{\prime }}{1+k_{l}^{\prime }}$$

where R is the radius of the Earth’s equator; θ and ϕ are latitude and longitude, respectively; $C_{lm}$ and $S_{lm}$ are the spherical harmonic coefficients of the gravity field; l and m are degree and order of the fully normalized associated Legendre function ${\bar{P}}_{lm}$; $h_{l}^{\prime }$ (horizontal direction) and $k_{l}^{\prime }$ (radial direction) are adopted load love numbers given by Farrell [1] and computed relative to the CM (center of the mass) of the solid Earth [7]. The significant raising of surface the water resource will deform the regional crust (including the seasonal and long-term trend deformation) and will be estimated in Section 3.1 and Section 3.2.

## 3. Results

### 3.1. Surface Mass Seasonal Changes

**Figure 4:**
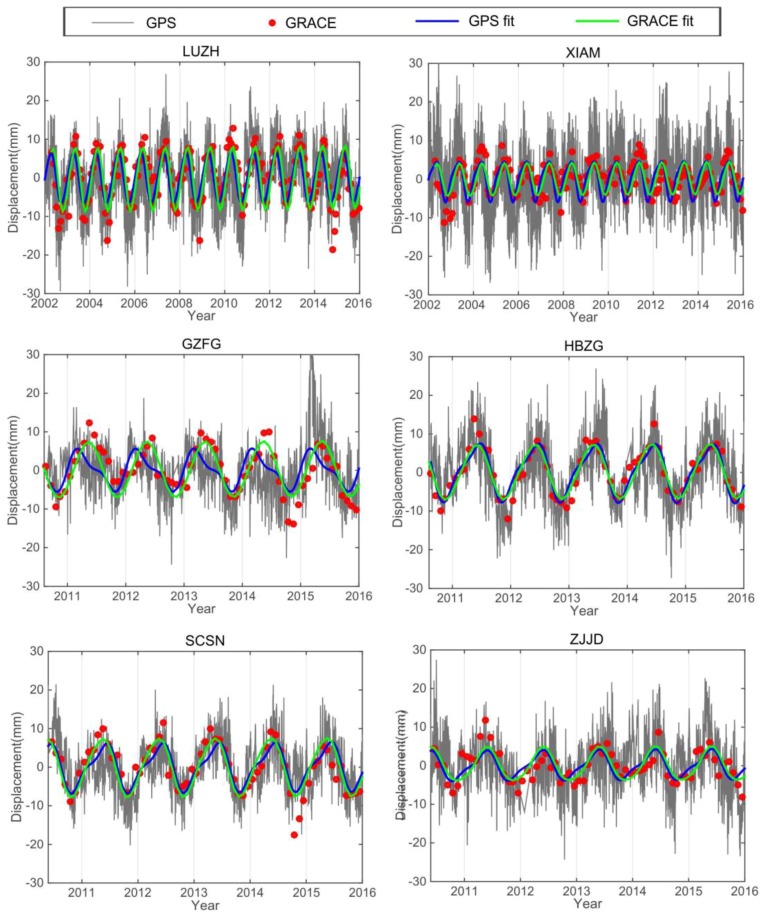
The seasonal variations comparison between de-trended continuous GPS and GRACE-modeled loading time series in Southern China, and example time series for sites LUZH, XIAM, GZFG, HBZG, SCSN and ZJJD (red dots in the location map). LUZH and XIAM have longer observational time than other four GPS sites. Blue and green solid lines are fitted by least square (annual plus semi-annual components) for GPS and GRACE, respectively.

We constrained the surface mass variations from CGPS and GRACE observations in SCB and surroundings. CGPS-derived seasonal loading patterns were compared with GRACE-derived surface seasonal mass loading variations, while the long-term trend was removed from above two time series. The annual and semi-annual terms were added for surface seasonal signals fitted by the least squares method. The amplitude and the trend coefficients and constants are obtained by the least squares, which take the annual period of $f_{1}$ = 1.04 (cycles per year, cpy), the semi-annual cycle $f_{2}$ = 2.08 (cpy) [39]. In addition to annual and semi-annual signals, 3.12 cpy, 4.16 cpy, 5.2 cpy and 6.24 cpy signals were also included in the GPS seasonal time series. However, the annual and semi-annual terms are the main components, indicating that the two signals are the largest contributions to seasonal signals. Figure 4 presents that the fitting by least squares of the six GPS-observed and GRACE-modeled time series in the SCB, showing seasonal surface mass loading oscillation consistency.

Figure 4 presents good consistency of the seasonal oscillation from GPS and GRACE measurements which demonstrate that the surface mass loading effects are the main contribution to the seasonal oscillation of GPS time series. In addition, the regional loading differences vary greatly in the different regions, as shown by the amplitude of seasonal signal in Figure 4.

The seasonal mass oscillations identified by GPS observations are largely due to surface loadings of the atmosphere, water, and ice mass origins. When estimating the vertical crustal deformations by GPS observations, the coherence between the seasonal oscillations derived from GPS and GRACE observations should be evaluated [40,41]. Here, we compared the variance between the GPS-derived seasonal signals and GRACE stacked-average seasonal signals. We derived mass loading changes by using GRACE measurements according to GPS sites location, and we stacked-average all GRACE-derived time series to compare the EOF decomposed signals of all GPS time series. First, we removed the GRACE-derived seasonal vertical deformations from the GPS-observed, de-trended height time series and computed the reductions in the weighted root-mean-square (WRMS) as follows:

(3) $$WRMS_{reduction}=\frac{WRMS_{GPS\_LS}-WRMS_{GPS\_LS-GRACE\_LS}}{WRMS_{GPS\_LS}}$$

(4) $$WRMS=\sqrt{\frac{\frac{n}{n-1}\sum_{i=1}^{n}\frac{{\left(P_{i}-P\right)}^{2}}{\sigma_{i}^{2}}}{\sum_{i=1}^{n}\frac{1}{\sigma_{i}^{2}}}}$$

where *n* is the number of days, $P_{i}$ is the estimate of the component on the i-th day, P is the weighted average of the component estimate over all days, and $\sigma_{i}$ is the formal error. Here, $WRMS_{reduction}$ = 1.0 indicates perfect consistency between GPS-observed and GRACE-modeled annual plus semi-annual displacements.

**Figure 5:**
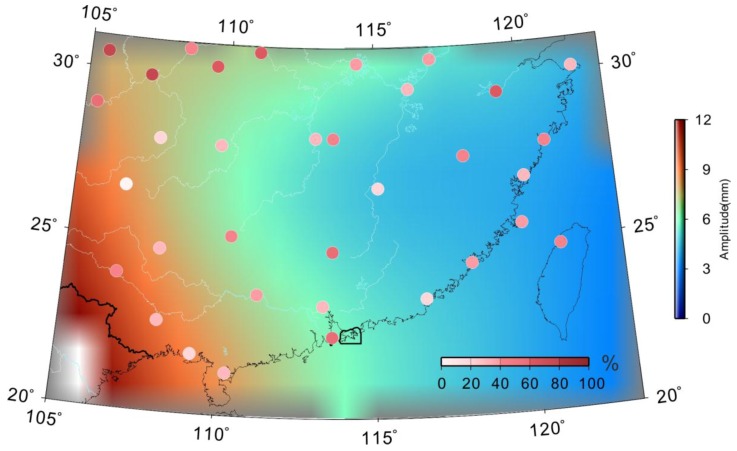
GRACE-derived mass loads amplitude for South China and *WRMS_reduction_* (weighted root-mean-squares) for each GPS sites when the GRACE-derived seasonal signals removed from GPS time series. The colored dots are the values of *WRMS_reduction_* for all GPS sites.

As shown in Figure 5, we computed the WRMS reduction ratio for 34 GPS stations by removing the GRACE-derived surface displacement from GPS-derived seasonal signals. The average WRMS reduction ratio of seasonal signals was 38% for 33 GPS stations. The consistency between the GPS common mode signals and GRACE stacked average seasonal signals which indicates the seasonal position oscillations in South China and surroundings. The mass loading identified by GPS seasonal displacement is mainly caused by surface Earth’s mass variations, including the hydrological, atmospheric and non-tidal ocean loads. However, some GPS sites show weak consistency with GRACE-derived surface displacement, due to the local mass anomalies (mainly caused by water resources) and spatial resolution of GRACE observation. We listed the relevant information in Table 1, including geodetic latitudes and longitudes of these stations, GPS- and GRACE-derived vertical long-term rates. The final vertical crustal velocity derived from GPS and GRACE is also given in Table 1 and will be discussed below.

Regional analysis at specific stations shows that the general vertical load sequence calculated from the time series of the GRACE model and GPS match each other well. However, the phase difference of the GRACE model and GPS time series is mainly relative to the Gaussian smoothing and de-striping applied for data processing of GRACE. In addition, the GRACE reaction is a large-scale mass change, and the GPS reaction is a point change, so there will be some differences in individual points.

In Figure 5, some GPS sites show low consistency with GRACE-derived loads due to local mass anomalies. In order to analyze the temporal–spatial consistency of seasonal mass loading deformation derived from GPS and GRACE measurements, we used the empirical orthogonal function (EOF) method to decompose the seasonal common mode of GPS time series. As a PCA, the EOF analysis decomposes the coherent spatio–temporal variability of a time-variable field into a linear combination of orthogonal “modes” of standing oscillation [22,42]. The detailed theory and procedure of the EOF algorithm are stated in Pan et al. [9] and Dong et al. [22].

**Figure 6:**
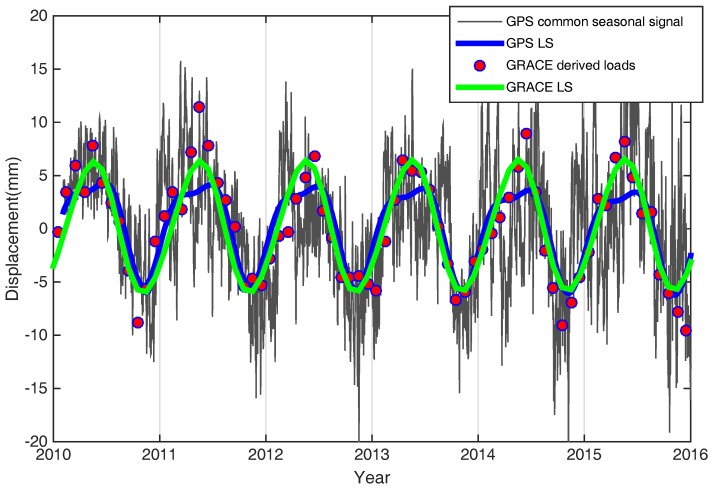
Comparison of empirical orthogonal function (EOF) decomposed first common mode of GPS stations and stacked average GRACE records are from 2010 to 2016. Gray lines (daily solutions) and red dots (monthly solutions) correspond to the GPS and GRACE results, which are fitted by least squares (LS) (i.e., the blue and green lines, respectively).

The EOF analysis was performed on the normalized GPS time series over the South China Block. The GRACE-derived loads corresponding to the GPS sites were stacked for an average seasonal signal, and then subtracted from the decomposed common mode signal of the GPS. The WRMS Reduction Ratio for all GPS stations is 0.87. Therefore, the different spatial scales between GPS and GRACE measurements can be explained largely by surface loading effects [43]. As shown in Figure 6, the consistency between the GPS-derived common mode signals and GRACE stacked average seasonal signals demonstrates that the seasonal position oscillations in South China are mainly caused by mass loading changes, including the hydrological, atmospheric and non-tidal ocean loads.

### 3.2. Vertical Crustal Deformation of SCB

**Figure 7:**
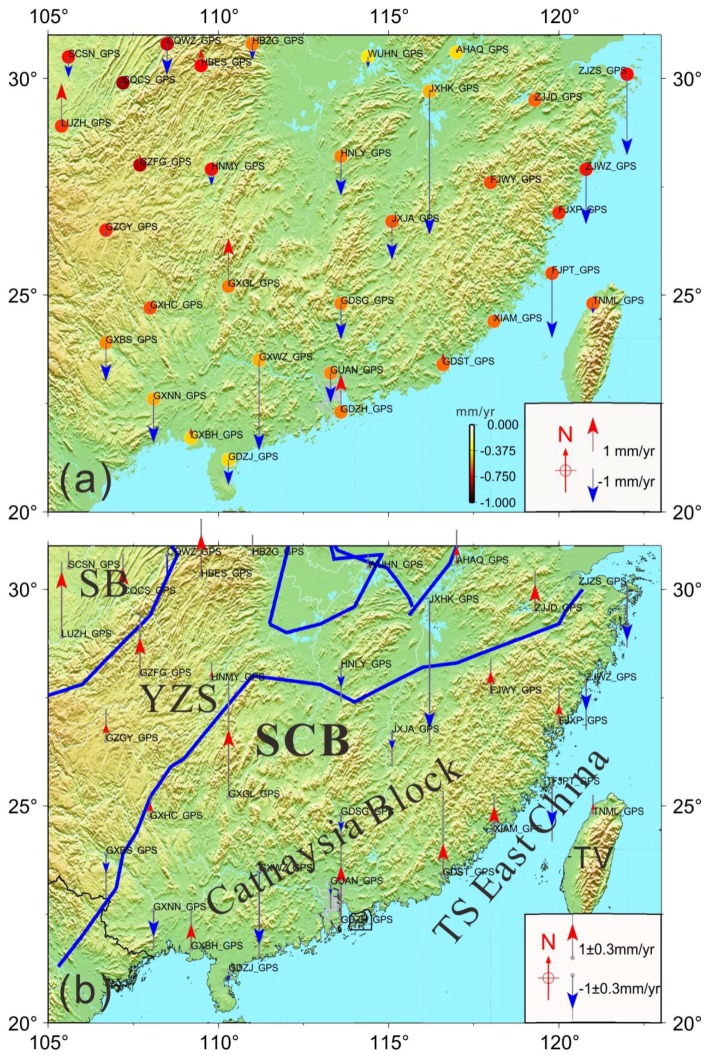
(**a**) The GRACE-RL5 was used to estimate the gravity in Southern China, spanning from April 2002 to January 2016. The dots denote the vertical deformations caused by surface loads corresponding to the GPS sites location. The vertical vectors are GPS-derived raw vertical rates; (**b**) Vertical crustal tectonic movement of SCB with the loading effects removed (by using GPS-derived rates to subtract GRACE-model loads). Red vectors denote subsidence uplift, and blue vectors denote subsidence. The gray lines behind arrows are the uncertainty of all sites. The blue boundary lines are tectonic lines in the South China Block. SB: Sichuan Basin; YZS: Yangtze Craton; SCB: South China Block; TS: Taiwan Strait.

As an elastic body, the Earth’s surface moves upward in response to a loss in loading and moves downward as the loading increases. As shown in Figure 7a, we computed the GRACE-derived long-term loadings using the trend from the CSR solutions corresponding to all CGPS sites used in this study. The least squares method was used to estimate the trend rates of all of the sites by considering the annual and semi-annual signals in the calculation. The surface deformation shows a subsidence rate of approximately −0.4 to −0.8 mm/year because of the increased loadings in SCB and surroundings. It shows a pattern of peaks at the Sichuan-Yunnan region, the west of South China, indicating that the maximum surface mass loads exist in this region corresponding to the GRACE-modeled total water storage secular variations.

We did not consider the influence of glacial isostatic adjustment (GIA) when calculating the long-term deformation of surface loads constrained from the GRACE solution. It is indicated that the above two factors have little contribution to the GRACE-modeled long-term trend gravity [7,8,9]. The vertical rate obtained from GPS deducting the load deformation caused by the GRACE-derived loading effects is the plate vertical tectonic movement of the crust. Figure 7b shows the vertical tectonic movement of the crust after removing the GRACE-modeled long-term trend mass loads from the GPS-derived vertical rates in the SCB. The crustal deformation is relatively stable compared with the active tectonic movement of the Tibetan Plateau, and there is no large active vertical tectonic movement in the SCB. The crust presents uplifting at rates of 0.2 mm/year to 2 mm/year in Sichuan-Yunnan rhombic block resulted from the compression of the surrounding crustal blocks. By contrast, sites undergoing subsidence are located in the center of the SCB, which could be caused by local crust and deep mass anomalies. The boundary of south coast line near TS East China show that a crustal uplifting at rate of approximately 1 mm/year may be relative to tectonic dynamic of sea–land interface.

## 4. Discussion

GRACE observations of mass variation and regional mass balance are limited by poor spatial resolution. The regional mass change will contribute to the Earth surface deformation due to mass loading effects. Here, we used the interpolation method to obtain the variation of loads deformation in northeast Tibet corresponding to the GPS locations by using GRACE observation. However, the consistency of spatio–temporal GPS and GRACE time series in South China indicating the seasonal oscillation is mainly caused by Earth surface loading effects. We obtained the vertical crustal deformation without considering the potential effects of GIA, which could contribute to both gravity changes and vertical motion. The GIA may have a significant impact on high mountains, such as the Himalayas and the Tibetan Plateau [25,44,45,46]. However, there is still a debate about whether there was a large ice sheet in the Himalaya and Tibetan Plateau during the last glacier period [47], let alone whether there was an ice sheet in South China. Additionally, the long-term gravity change related to tectonic isostatic equilibrium should be of little consequence to the cryospheric effect. Therefore, we ignored the effect of GIA on gravity changes when we used GRACE data to obtain the mass change. The corrected vertical velocity constrained by GPS and GRACE measurements was mainly caused by the crustal tectonic movement [9].

Other long-term geophysical effects (i.e., the frequency dynamic of Earth inner core and mantle anelasticity) may contribute the Earth surface variation and long-term trend deformation, which will bias the precision of geodetic observations [48]. Recently, it has been confirmed that the observed rapid polar motion since 2005 has resulted in a large-scale elastic radial deformation of the Earth in some local places [49,50]. This ensures that the geo-potential field is aligned to the long-term mean pole within the present geodetic secular observation accuracy. The geodetic vertical velocity fields, for instance based on GPS and GRACE observations, are affected by rapid changes of polar motion [47]. The GPS processing of the deformation associated with such deviations in polar motion from its longer-term path is not corrected through the pole tide model, which addresses only a periodic deviation away from a time variable reference pole position. In addition, we did not consider the contributions of thermal expansion of monuments and nearby bedrock to observed GPS height changes reported by Yan et al. [51], which may be relative to the basement condition of GPS station sites.

## 5. Conclusions

We used data from 33 continuous GPS sites to investigate the surface seasonal mass changes and vertical crustal movements in and around South China from 1999–2016. The uncertainly of GPS velocity were improved by removing common mode errors (CME) from time series. We presented above the mass rate in and around South China estimated from GRACE (CSR RL05) for the period of April 2002 to January 2016. The seasonal mass oscillations will contribute to surface loading deformation due to redistributions of the land mass loads, as observed by CGPS and GRACE measurements in South China. The correlation was analyzed between GPS-observed and GRACE-modeled surface seasonal mass changes (see Figure 3). From the analysis results it can be seen that the seasonal variation of GPS and GRACE is consistent, which demonstrates that the seasonal variation of GPS is mainly caused by the surface loads deformation.

In addition to the dominating seasonal signals, trends in GRACE-modeled mass changes are evident and can be attributed to gradual accumulations of crustal materials caused by surface terrestrial water storage anomalies. We modeled the surface mass loads by GRACE measurement and removed it from the corresponding GPS sites (Figure 4). Besides the seasonal changes, the loading effects will also have a long-term impact (Figure 7a). In the calculation of crustal deformation and tectonic movement of the surface after the impact load deformation of the crust, this method provides a good way for the GPS new vertical field to be seen in the vertical crust deformation of the SCB. The uplift and subsidence of South China, indicated by the GPS after removing the GRACE-derived long-term rate, is due to secular changes of regional tectonic origin (Figure 7b).

## Figures and Tables

**Table 1:** The information of GPS stations with their vertical velocities, and GRACE-modeled uplift rates.

Site	Lat. (°)	Long. (°)	Duration	GPS-Derived Vertical Velocity (mm/year)	GRACE-Modeled Uplift (mm/year)	Tectonic Vertical Rate (mm/year)	WRMS Reduction (%)
AHAQ	117.0	30.6	2010–2016	0.101 ± 0.447	−0.424 ± 0.067	0.525 ± 0.452	35
CQCS	107.2	29.9	2010–2016	−0.092 ± 0.400	−0.846 ± 0.056	0.753 ± 0.404	74
CQWZ	108.5	30.8	2010–2016	−0.918 ± 0.636	−0.794 ± 0.058	−0.124 ± 0.639	44
FJPT	119.8	25.5	2010–2016	−1.892 ± 0.408	−0.640 ± 0.049	−1.252 ± 0.411	37
FJWY	118.0	27.6	2010–2016	0.019 ± 0.352	−0.642 ± 0.054	0.661 ± 0.356	43
FJXP	120.0	26.9	2010–2016	−0.097 ± 0.503	−0.687 ± 0.064	0.589 ± 0.507	28
GDSG	113.6	24.8	2010–2016	−1.058 ± 0.488	−0.560 ± 0.056	−0.498 ± 0.491	60
GDST	116.6	23.4	2010–2016	0.348 ± 1.525	−0.596 ± 0.067	0.944 ± 1.526	15
GDZH	113.6	22.3	2010–2016	1.112 ± 0.631	−0.568 ± 0.057	1.680 ± 0.633	53
GDZJ	110.3	21.2	2010–2016	−0.740 ± 0.346	−0.429 ± 0.058	−0.311 ± 0.351	27
GUAN	113.3	23.2	1999–2016	−0.881 ± 0.388	−0.553 ± 0.062	−0.328 ± 0.393	28
GXBH	109.2	21.7	2010–2016	0.287 ± 0.433	−0.441 ± 0.035	0.728 ± 0.434	12
GXBS	106.7	23.9	2010–2016	−1.122 ± 0.709	−0.542 ± 0.062	−0.580 ± 0.712	46
GXGL	110.3	25.2	2010–2016	1.413 ± 1.442	−0.571 ± 0.066	1.984 ± 1.443	47
GXHC	108.0	24.7	2010–2016	−0.136 ± 0.344	−0.631 ± 0.081	0.495 ± 0.353	22
GXNN	108.1	22.6	2010–2016	−1.296 ± 0.338	−0.493 ± 0.064	−0.803 ± 0.344	22
GXWZ	111.2	23.5	2010–2016	−2.662 ± 0.400	−0.489 ± 0.073	−2.173 ± 0.406	34
GZFG	107.7	28.0	2010–2016	0.294 ± 0.787	−0.822 ± 0.071	1.116 ± 0.790	11
GZGY	106.7	26.5	2010–2016	−0.177 ± 0.398	−0.691 ± 0.064	0.514 ± 0.403	09
HBES	109.5	30.3	2010–2016	0.486 ± 0.442	−0.748 ± 0.064	1.234 ± 0.447	64
HBZG	111.0	30.8	2010–2016	−0.469 ± 0.464	−0.571 ± 0.077	0.101 ± 0.470	67
HNLY	113.6	28.2	2010–2016	−1.161 ± 0.411	−0.559 ± 0.049	−0.602 ± 0.413	46
HNMY	109.8	27.9	2010–2016	−0.482 ± 0.277	−0.734 ± 0.053	0.251 ± 0.282	26
JXHK	116.2	29.7	2010–2016	−4.210 ± 0.486	−0.493 ± 0.068	−3.717 ± 0.491	20
JXJA	115.1	26.7	2010–2016	−1.134 ± 0.476	−0.618 ± 0.055	−0.516 ± 0.479	15
LUZH	105.4	28.9	1999–2016	1.256 ± 0.251	−0.668 ± 0.072	1.924 ± 0.261	55
SCSN	105.6	30.5	2010–2016	−0.607 ± 0.350	−0.726 ± 0.073	0.119 ± 0.357	73
TNML	121.0	24.8	1999–2016	−0.300 ± 0.240	−0.619 ± 0.065	0.319 ± 0.248	40
WUHN	114.4	30.5	1999–2016	−0.321 ± 0.271	−0.403 ± 0.051	0.081 ± 0.276	39
XIAM	118.1	24.4	1999–2016	0.170 ± 0.283	−0.592 ± 0.059	0.762 ± 0.289	38
ZJJD	119.3	29.5	2010–2016	0.178 ± 0.364	−0.601 ± 0.068	0.779 ± 0.371	67
ZJWZ	120.8	27.9	2010–2016	−1.616 ± 0.547	−0.719 ± 0.055	−0.897 ± 0.550	42
ZJZS	122.0	30.1	2010–2016	−2.380 ± 0.180	−0.738 ± 0.058	−1.642 ± 0.189	27
